# Development and Piloting of a Patient-Centered Report Design for Stress Myocardial Perfusion Imaging Results

**DOI:** 10.1001/jamanetworkopen.2021.21011

**Published:** 2021-08-20

**Authors:** Krishna K. Patel, Carole Decker, Christina M. Pacheco, Christine Fuss, Illham Boda, Kensey L. Gosch, Arthur I. McGhie, Randall C. Thompson, Brett W. Sperry, Timothy M. Bateman, John A. Spertus

**Affiliations:** 1 Saint Luke’s Mid America Heart Institute, Kansas City, Missouri; 2 University of Missouri–Kansas City School of Medicine, Kansas City, Missouri; 3 University of Kansas School of Medicine, Kansas City, Kansas

## Abstract

**Importance:**

The management of coronary disease epitomizes the call to better engage patients in shared medical decision-making. Myocardial perfusion imaging (MPI) is the foundation of diagnosis, risk stratification, and subsequent therapy; however, MPI reports are currently interpretable by specialists but not patients.

**Objective:**

To design and test a patient-centered report for stress MPI test results.

**Design, Setting, and Participants:**

This qualitative study of outpatients who underwent an MPI stress test and clinicians used a mixed methods approach. Phase 1 (December 2018 to July 2019) used qualitative methods to design a patient-centered reporting tool, with 5 focus groups with 36 patients and 2 focus groups with 27 clinicians. Phase 2 (June to September 2019) consisted of pilot testing the reporting tool with feedback from a structured survey given to patients who received MPI reports before and after implementing the tool.

**Main Outcomes and Measures:**

Key themes around patient experiences with the current MPI reporting and their desire for a more useful report were identified, which led to a sample reporting tool after serial iterations with feedback. Differences in patient knowledge and engagement were assessed between patients before and after implementation of the new reporting tool using χ^2^ tests.

**Results:**

From patient focus groups (26 patients; mean [SD] age, 66.3 [9.6] years, 9 [35%] women), 3 themes on the inadequacies of current MPI reporting were identified: (1) inconsistent delivery of results, (2) use of medical jargon, and (3) unclear posttest course. We identified 5 themes for a more patient-centered MPI report: desire for written information, discussion of the report with medical personnel, presentation of results in simple language with use of visual graphics, comparisons with normal results, and personalized risk estimates. In a pilot survey with 123 patients split into a pre-implementation group (69 patients; mean [SD] age, 68.2 [8.5] years; 27 [51%] women) and a postimplementation group (54 patients; mean [SD] age, 66.4 [8.7] years; 30 [56%] women), the patient-centered report led to more patients reading the entire report (45 [83%] vs 46 [67%]; *P* = .04) and improved knowledge of future risk of cardiac events (41 [76%] vs 20 [29%]; *P* < .001). There was also a numerically higher percentage of patients who found the report easy to read (45 [83%] vs 44 [68%]; *P* = .05) and understand (42 [78%] vs 43 [66%]; *P* = .16), although these results were not statistically significant.

**Conclusions and Relevance:**

This study identified key elements of a patient-centered report design for stress MPI test results, which improved patient engagement and knowledge. These preliminary data support further implementation and study of a more patient-centered MPI report.

## Key Points

**Question:**

What information do patients want after undergoing a cardiac stress test?

**Findings:**

In this qualitative study of 36 patients in focus groups and 123 patients in a pilot test, 5 broad themes that could be incorporated into myocardial perfusion imaging reporting to better meet patients’ needs emerged: (1) providing written information, (2) discussing report with medical personnel, (3) presenting results in simple language with graphics, (4) providing comparisons with normal results, and (5) providing personalized risk estimates, especially in the context of posttest management. Incorporating these themes into a sample patient-centered report was associated with a greater proportion of patients reading the reports and understanding their future cardiac risk.

**Meaning:**

Reports incorporating key elements identified in this study could lead to improved patient satisfaction and, potentially, better care and outcomes by increasing patients’ understanding of and engagement with treatment.

## Teaser

This qualitative study designs and tests a patient-centered report for stress myocardial perfusion imaging test results.

## Introduction

Myocardial perfusion imaging (MPI) has a central role in diagnosis, risk stratification, and subsequent management for patients with known or suspected coronary artery disease (CAD), but the technical reports are geared toward referring specialists, not patients. To improve the patient-centeredness of care, MPI reports should clearly communicate their findings to both clinicians and patients. The former group is important because referring clinicians with less knowledge about the specifics of the imaging study often order these studies and need to communicate the results to their patients. With the recent implementation of the 21st Century Cures Act,^1^ patients now have immediate access to their health records, including MPI reports, underscoring the importance of improving their interpretability for patients to a provide basic understanding of the results, prognosis, and potential posttest management strategies. There is significant variability in the management of patients after MPI.^2^ Patient-centered MPI reports could potentially facilitate shared decision-making between patients and their physicians, thereby reducing variability and increasing appropriate posttest management and resource utilization. In the current study, we used a mixed methods approach to define key elements and then design and pilot test a patient-centered MPI report.

## Study Methods

This was a multiphase mixed methods qualitative study. Phase 1 used qualitative methods to understand the value of MPI reports to patients while gathering feedback on iterative modifications to further understand how these reports could best support decisions for future treatment. This process was replicated with a panel of clinicians. After all feedback was collected, a final draft tool was created. In phase 2, we surveyed patients before and after initiating the new MPI report to assess patients’ knowledge, satisfaction, and engagement. The study was approved by the Saint Luke's Hospital of Kansas institutional review board. All focus group participants signed written informed consent, and all pilot test participants provided verbal informed consent. This study followed the Consolidated Criteria for Reporting Qualitative Research (COREQ) reporting guideline.^3^

### Focus Groups

Participation in the focus groups required that patients had received a stress test in the past 12 months at the study site and had no history of CAD at time of testing. We used purposeful sampling to randomly invite 143 patients to participate in focus groups. We sought a broad representation by age, sex, patient-reported race, education, and clinical setting. We conducted 5 focus groups until saturation was achieved (36 patients). We invited 9 participants from the focus groups to provide feedback on the evolution of the tool’s design. The focus groups were led by trained and experienced facilitators, which included 2 clinical research nurses (C.D. and C.F.) and a health services researcher (C.M.P.). Using a phenomenology framework,^4^ all focus groups followed a structured moderator’s guide (eTable in the Supplement). Feedback regarding report content and layout was collected prior to implementation. Two clinician panels with 27 participating clinicians were held to report patient feedback and elicit clinician input. Clinicians provide verbal informed consent.

### Iteration of the MPI Report Content

As the patient focus groups progressed, we designed and iteratively revised the patient-centered MPI report, presenting changes to subsequent groups. For this study, the content targeted patients with no history of prior myocardial infarction or coronary revascularization.

**Figure 1.**
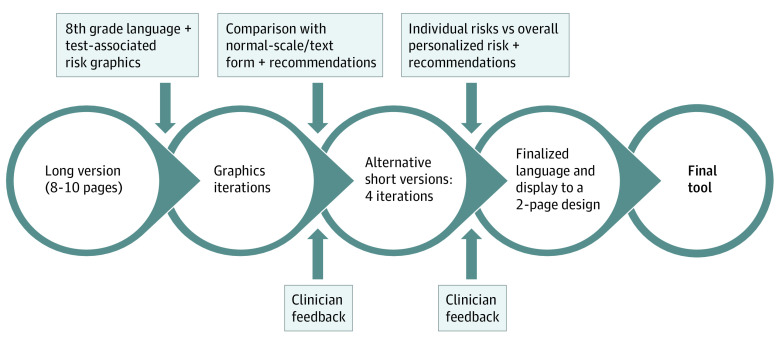
Evolution of the Patient-Centered Tool

In phase 1 (December 2018 to July 2019), 4 different iterations of the patient tool were presented. The initial report was assessed at a grade 13.6 reading level using Flesch-Kincaid readability statistics. The final report was assessed at a grade 8.3 reading level and included figures and graphs for visual appeal and to support understanding. Clinicians provided input twice, 3 months apart (Figure 1).

Decision support material was structured in the following manner, with modifications based on focus group feedback: (1) explanation of test results in lay language and (2) presentation of individualized or population-based risks and benefits with alternative treatments (eFigure 2 in the Supplement). Given that there were no previously published validated risk prediction models, we used placeholders developed from our institutional database.^5,6,7,8,9^ The tool was cross-checked for inclusion of characteristics defined by the International Patient Decision Aids Standards Collaboration.^10^ The penultimate tool was presented to a panel of expert physicians, nurses, and advanced practice clinicians to obtain feedback regarding accuracy, ease of interpretation, and layout. Cognitive debriefing of the final content and layout of the new report was performed in the final focus group, which consisted of 9 participants who had been part of early focus groups.

### Focus Group Analysis

The focus group discussions were recorded and transcribed verbatim. The study team, composed of a cardiology fellow (K.K.P.), a medical student (I.B.), a health services researcher (C.M.P.), and 2 clinical research nurses (C.D. and C.F.), coded the text independently of each other. Transcripts were examined for specific patterns and themes, and statements were formally coded. On identifying the codes, a visual display of key themes, responses, and attributes were created. A taxonomy of themes was created to assign the final coding of the raw narrative data. A similar process was conducted with the clinician panel transcripts. These steps were performed according to the highest methodological standards available for doing qualitative research.^11,12,13,14^ Following the individual coding process, the team reviewed conclusions drawn from the data. Conclusions were evaluated in the context of all of the data and were modified when case discrepancies were identified.

### Pilot Test

From June to September 2019, patient-centered MPI reports were tested among 123 patients (target sample size, 100) with no known history of CAD presenting for clinically indicated MPI at our imaging laboratory using a structured survey to assess readability, understanding, and patient satisfaction. To ensure consistent patient access, all reports were mailed to patients with a cover letter explaining voluntary participation in a telephonic survey. No change was made in the usual standard of care received by patients except for the mailing of the reports. We mailed 129 standard test reports to patients, and 69 patients participated in the pre-intervention survey. We then mailed 67 postintervention reports (standard report with a personalized 2-page addendum) to patients, and 54 patients participated in the follow-up survey. A follow-up telephone survey was conducted by the research team between 7 and 14 days after the reports were mailed.

### Statistical Analysis

Patient characteristics and survey responses were compared between the pre-intervention and postintervention groups with *t *tests for continuous variables and χ^2^ or Fisher exact tests for categorical variables. A 2-sided *P* < .05 was considered significant. All analyses were performed using SAS version 9.4 (SAS Institute).

## Results

### Qualitative Insights Provided by Patient Participants

Characteristics of the 26 individuals who participated in 1 focus group (mean [SD] age, 66.3 [9.6] years, 9 [35%] women) are shown in Table 1. Based on the participant report, 7 (27%) had a normal result, 12 (46%) had an abnormal result, and 6 (23%) did not know their MPI result at the time of their focus group. Although attempts were made to increase diversity, nearly all patients (24 [92%]) were White individuals.

**Table 1.** Characteristics of Patient Focus Group Participants^a^

Characteristic	Participants, No. (%) (N = 26)
Age, mean (SD), y	66.3 (9.6)
Women	9 (35)
Men	17 (65)
Caucasian	24 (92)
Education	
High school education or less	5 (19)
Vocational school or some college	6 (23)
4-y College degree	6 (23)
Postgraduate education	9 (35)
Living location	
Urban	6 (23)
Suburban	17 (66)
Rural	3 (12)
Time of most recent stress test	
<6 mo	14 (54)
>6 mo to 1 y	10 (39)
>1 y	2 (8)
Patient-reported stress test result	
Normal	7 (27)
Abnormal	12 (46)
Do not know	6 (23)

^a^ These data exclude the 9 repeat participants and 1 participant who did not fill out the demographic survey form.

**Figure 2.**
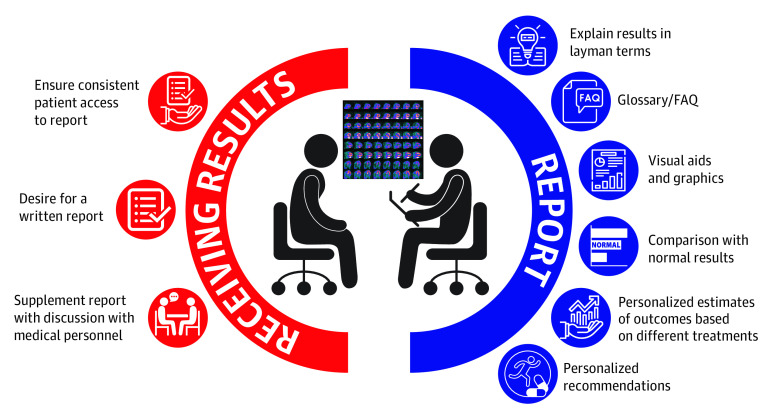
Components of a Patient-Centered Stress Test Report, Incorporating Main Themes Identified From Patient Focus Groups FAQ indicates frequently asked questions.

Results from patient focus groups were coded into 2 main categories: (1) current reporting and (2) patient-centered design. The 2 main categories had 8 subthemes desired by patients (Figure 2).

#### Current Reporting

Three themes emerged related to patients’ experience with their prior, clinically indicated, MPI study reports. First was inconsistent delivery of results. It was quickly learned that patients received the results from multiple and inconsistent settings, including direct access by the patient through their patient portal and/or electronic medical record; via telephone call; a paper copy through the mail; or, as 1 patient stated, “They never did call and I never got a letter. I didn’t get anything.”

Second was medical jargon, which led to poor understanding, often requiring patients to do research on the internet. One patient participant stated, “It’s just very medical termy. I feel like this is definitely more so an internal medical record as opposed to a report that you would hand a patient. I understand about 10% of this.” Another patient explained that they “spend a lot of time on Google trying to get the definitions of each of the words.”

The third theme was an unclear posttest course**,** which led to a desire for more information. One patient said, “After a test, everybody’s got questions.” Another patient summarized it by saying, “I kept wishing they would send somebody in to tell me what was what and what you should do afterwards.”

#### Patient-Centered Design

Five themes emerged related to the patients’ description of a better potential patient-centered design. First was a desire for written information. A patient stated, “But I would like this afterwards to have something to go back to, if I forgot what they said.” Another said, “I have a better time if it’s in front of me instead of a phone call. Because then if there’s something I don’t understand and I ask them, okay, what about this.”

A second theme was that they wanted a discussion with medical personnel with the written report. One patient said, “With communication anyway, I think in order for someone to understand something, you have to communicate it in several different ways.”

A third theme was to make it simple and visual. A strong theme articulated by patients was that they wanted a tool in lay terms, accompanied by a glossary or a frequently asked questions section. One patient said, “It’s like you do get confused.… And it would be nice if there was a way to turn that stuff into layman terms.” Another patient said that having both kinds of language (clinical terminology and layman terms) was valuable: “The nomenclature is important so that when we want to take matters in our own hands, we know what to look for.” They also explicitly articulated a desire for visual aids and graphics to depict risk. One patient said, “A picture is worth a thousand words. If you show me a picture, I’m going to remember it better than I’m going to remember all of this.”

The fourth theme was to provide comparison with normal results. One patient said, “I couldn’t figure out what the things meant because most of the results were just factual results. They weren’t compared to normal.” Another patient said, “When you get a blood test and your blood results and it gives you like glucose 113, normal should be 80 to 100 or something like that. You give the value, and you give a range, and you give some comparison between normal and abnormal.” Another patient said, “It would be nice to know what an absolutely healthy person scored.”

A fifth theme was to provide personalized risk, especially in the context of an intervention. A patient said, “I kind of think, this one-size-fits-all type thing, it’s not the way to go. I mean, based on your age and gender and in this group, this what’s expected that could happen—that would be better.” Another patient put it very plainly, “But risk if you do what? Common sense tells you that it would be due to treatment, so say, if I take baby aspirin, or if I do this, this is my risk.”

### Qualitative Insights Provided by Clinician Participants

Two clinician panels were interspersed with the patient focus groups to collect feedback on the evolving tool. It included a total of 27 clinicians, including internists, cardiologists, nurses, residents, and research and cardiology fellows. Results from the clinician panel were coded into 3 main categories: increased clinical burden, information overload, and inclusion of risk models.

#### Increased Clinical Burden

Clinicians were concerned about the increase in time required to use the new report format, especially if it generated a lot of questions. Some suggested that telephone visits or virtual visits with the physician or advanced practice clinicians to discuss the stress test results could be a potential solution. One clinician shared, “I am just worried about the practical aspect of [it] … Right now, it’s not feasible for us to see all these patients back. And if the [report] generate[s] a lot of questions, the nurses can’t handle it.” Another added, “Ideally [the stress-test results] should be a physician-patient discussion, but in reality, it’s impossible to do in our practice.”

#### Information Overload

Two themes emerged related to information overload. First was that providing too much information may cause patient harm. One clinician stated, *“*Talking about ACE [angiotensin-converting enzyme] inhibitors and β-blockers are going to go over [patients’] heads. And talking about procedures like angiography, you’re probably going to make more people concerned than comfort them*.*” Another shared, “I also think that this is going to open a lot more questions than it’s going to solve.”

The second theme was a need to simplify the report and only provide necessary information. One clinician added, “We have to simplify this, synthesize it into 1 page with some graphics, with some risk estimate.… Other than that, you start losing, getting conflicting information and basically some things that we don’t think [patients] need to understand at this point in time.”

#### Inclusion of Risk Models

Clinicians liked the idea of including information risks by intervention. A participant shared, “It would be helpful to have information about the extent to which interventions would or would not mitigate the risk. … Where I plug in patient data—I start them on an ACE and the risk goes down by this much or something.” Another stated, “I think it would be helpful to have information about the extent to which interventions would or would not mitigate the risk.” “The interpretation of an abnormal stress test can vary from 1 patient with a known disease to somebody without disease,” another clinician shared.

### Pilot Test Survey

After consolidating the information from phase 1, an initial prototype report was built, as shown in eFigure 2 in the Supplement. We then pilot tested the new report as an appendix to the current standard-of-care reports. Overall, 123 patients split into a pre-implementation group (69 patients; mean [SD] age, 68.2 [8.5] years; 27 [51%] women) and a postimplementation group (54 patients; mean [SD] age, 66.4 [8.7] years; 30 [56%] women). Participant characteristics are shown in Table 2.

**Table 2.** Characteristics of the Pilot Survey Participants

Characteristic	Participants, No. (%)		*P* value
	Pre-implementation (n = 69)	Postimplementation (n = 54)	
Age, mean (SD), y	68.2 (8.5)	66.4 (8.7)	.26
Women	35 (51)	30 (56)	.63
Men	34 (49)	24 (44)	
Race			
White	58 (84)	47 (87)	.64
Black	9 (13)	4 (7)	
Other	2 (3)	3(6)	
Education level			
High school education or less	22 (32)	14 (26)	.65
Vocational school or some college	25 (36)	17 (32)	
4-y College degree	13 (20)	10 (19)	
Postgraduate education	8 (12)	13 (24)	
Patient-reported stress test result			
Normal	47 (68)	39 (72)	.86
Abnormal	14 (20)	9 (17)	
Do not know	8 (12)	6 (11)	

**Figure 3.**
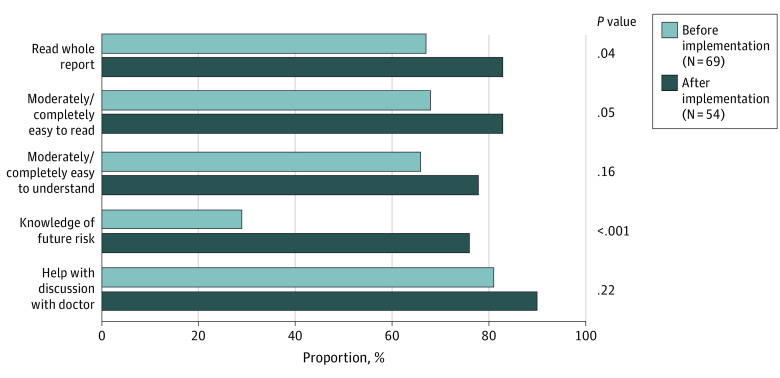
Results of the Pilot Survey Comparing 69 Patients Who Received the Current MPI Report With 54 Patients Who Received the Personalized, Patient-Friendly Addendum With the Current MPI Report

After implementation of the patient-centered report, a higher proportion of patients reported that they had read the entire report (45 [83%] vs 46 [67%]; *P* = .04). Furthermore, after implementation, a greater proportion of patients reported knowledge of their future risk of cardiac events (41 [76%] vs 20 [29%]; *P* < .001). There was also a numerically higher percentage of patients found the report easy to read (45 [83%] vs 44 [68%]; *P* = .05) and understand (42 [78%] vs 43 [66%]; *P* = .16), and more noted that the report will help them with their discussions with physicians (46 [90%] vs 55 [81%]; *P* = .22); however, these results were not statistically significant (Figure 3).

## Discussion

Current MPI reports are written at a very technical level, which may be difficult for referring physicians and patients to understand. To improve communication after stress testing, we conducted a mixed-methods study to understand the elements of a patient-centered stress MPI report, based on perspectives from patients and referring clinicians, and then pilot tested a prototype supplement to current MPI reports. Participants identified several key themes for an ideal patient-centered report: access to a written report supplemented by discussion with medical personnel, description of results in simple language, use of visual aids, presentation of results in the context of normal results, and a description of personalized risk with recommendations. The patient-centered report, created with stakeholder input, improved readability and facilitated better understanding of results and knowledge of future risk.

To our knowledge, this is the first assessment of elements of a patient-centered MPI reporting experience from a patient’s perspective. While our study focused on stress MPI reporting, the key themes identified are potentially applicable to other imaging modalities. Our results provide a potential strategy for imaging laboratories to develop patient-centered reports and could serve as a reporting template for imaging modalities beyond MPI.

Patients described inconsistent access to MPI reporting. Despite multiple government initiatives to empower patients’ access to their medical records,^15^ most patients did not access or did not believe they had access to their reports. Overwhelmingly, patients reported the desire to have a written report supplemented by a conversation with medical professionals to understand results, discuss management strategies, and discuss potential impacts and risk of cardiac events.

There is evidence supporting the use of decision support tools outside of nuclear cardiology.^16,17^ In our study, patients and clinicians showed a strong interest in having access to a decision support tool that provides a personalized patient risk score based on test results. Discussion of posttest treatment options, including invasive angiography and/or medical management, were viewed as valuable. Development and validation of robust personalized risk prediction models for patients undergoing MPI testing, including patient and test characteristics (eg, from the recent ISCHEMIA trial^18,19^), to project outcomes with intervention vs medical management after MPI is a key actionable item identified from our study.

A novel aspect of our work is the multistakeholder design. We engaged patients, nursing and support staff, and referring physicians in the process of designing the decision support tool to increase their relevance and impact. A reporting infrastructure built incorporating the themes identified from this study can fulfill various key requirements of Merit-Based Incentive Payment System through patient education, care coordination, use of decision support tools, and the collection of patient-reported outcomes.^20^

### Limitations

This study has limitations. Qualitative research is exploratory in nature and may have limited generalizability. To mitigate this, we sought input from a broad spectrum of patients and from physicians at different levels of training, from different specialties, and working in different practice settings. Despite our efforts, we included few people who identified as Black, Hispanic, or another racial/ethnic group, and further work is needed to ensure their perspectives are better represented. Throughout the process, we made efforts to reconcile differences in patient and clinician perspectives, taking both into account as we iteratively refined the tool. Importantly, despite the interests of both patients and clinicians in risk prediction models, few are currently available for new onset CAD. Accordingly, the risk prediction models used in this project were placeholders developed from our internal patient databases. These should be replaced with risk models from randomized clinical trial data when available. Referring physician feedback regarding changes in patient engagement and adherence to treatment could not be obtained for the pilot survey and should be tested in future studies.

## Conclusions

In this study, we identified essential elements for patient-centered MPI reporting using patient focus groups. We provided preliminary data that including patient-centered reporting with decision support within an MPI report could improve patient knowledge of future risks, treatment options, and understanding of test results. This can potentially lead to reduced variability in care, improved patient satisfaction, and better care coordination by the imaging cardiologist. Future directions of our work include validation of the tool in a larger, more diverse population across different health care systems; extending to other patient populations, such as those with prior CAD; implementation of patient-centered reports in routine clinical workflow; and an assessment of the tool’s association with posttest management, patient adherence to treatment, and long-term outcomes.

## Notes

Supplement. **eTable. **Sample Open-Ended Questions From the Interview Guide **eFigure 1. **Example of a Current Myocardial Perfusion Imaging Report in Our Imaging Laboratory **eFigure 2. **Sample Design of Patient-Centered Tool Sent as an Addendum to the Structured Patient Report
