# Error in Byline

**DOI:** 10.1001/jamanetworkopen.2021.34403

**Published:** 2021-10-12

In the Original Investigation titled “Development and Piloting of a Patient-Centered Report Design for Stress Myocardial Perfusion Imaging Results,”^1^ published August 20, 2021, there was an error in the byline. Ilham Boda’s name was misspelled. This article has been corrected.^1^
