# Supplementary material for: Development and Piloting of a Patient-Centered Report Design for Stress Myocardial Perfusion Imaging Results
Source: JAMA Netw Open. 2021 Aug 20;4(8):e2121011. doi: 10.1001/jamanetworkopen.2021.21011 (PMC8379654; doi:10.1001/jamanetworkopen.2021.21011)
Supplement: Supplement. — eTable. Sample Open-Ended Questions From the Interview Guide eFigure 1. Example of a Current Myocardial Perfusion Imaging Report in Our Imaging Laboratory eFigure 2. Sample Design of Patient-Centered Tool Sent as an Addendum to the Structured Patient Report [file jamanetwopen-e2121011-s001.pdf]

Supplemental Online Content

Patel KK, Decker C, Pacheco CM, et al. Development and piloting of a patient-centered report design for stress myocardial perfusion imaging results. *JAMA Netw Open.* 2021;4(8):e2121011. doi:10.1001/jamanetworkopen.2021.21011

eTable. Sample Open-Ended Questions From the Interview Guide

eFigure 1. Example of a Current Myocardial Perfusion Imaging Report in Our Imaging Laboratory

eFigure 2. Sample Design of Patient-Centered Tool Sent as an Addendum to the Structured Patient Report

This supplemental material has been provided by the authors to give readers additional information about their work.

© 2021 Patel KK et al. *JAMA Network Open.*

eTable. Sample Open-Ended Questions From the Interview Guide

Tell us about your experience when you had your stress test
PART I – STRESS TESTING EPISODE<br><br>• Do you know what the test showed?<br>• How was your experience in receiving the results of your stress test? Can you tell us about how you received the results of your stress test? What did you like or not like about that?<br><br>Probes for facilitator:<br>o Did you get the stress test report with the results?<br>o Did the report provide meaningful information to you? Was it clear to you what the test showed?<br>o Did you review it with anyone, did you have any problems understanding the report and what it meant?<br>o Did you know what the treatment options were after you received your stress test results? Was it clear to you what your treatment options were?<br>o How involved were you in the decision regarding treatment after your stress test? Did you have a chance to choose between treatments? Did you weigh in on the decision? Seek another opinion?
PART 2- DESIGNING REPORT<br><br>• What information would you like with your stress test results to help you understand it better and to help you make treatment decisions after the test?<br>• Facilitator note: Show current tool (for focus groups after the first one, show a mock-up of new report along with the original report) This is a sample of the stress test report we send to doctors and patients currently.

© 2021 Patel KK et al. *JAMA Network Open.*

- How meaningful is this report to you? Are you able to understand all parts of this report?
- How can we make this better? (PROBES: What specific information would you wish to know from your stress test report? Would you like to receive recommendations in your stress test report about possible treatments based on your results?)
- This is a sample of the new report we are creating based on your feedback. How do you feel about this report? How can we make it better?
- Looking back on whole experience of getting the results of your stress test, is there anything about the process that could be improved upon?
- Would you like to review the final content of the report we create after incorporating your feedback? What would be the best way to contact you to check-in with you regarding the results of this study?
- At the end of the study, we want to tell patients what we learned, what would be the best way for us to do that?

© 2021 Patel KK et al. *JAMA Network Open.*

eFigure 1. Example of a Current Myocardial Perfusion Imaging Report in Our Imaging Laboratory

ICANL
Nuclear Cardiology
Accredited Nuclear Cardiology Laboratory

Saint Luke's Health System

REST/STRESS REGADENOSON RUBIDIUM-82 PET/CT REPORT
WITH CALCIUM SCORING

**PATIENT**	**TEST DATE:**
**GENDER**	**AGE O**
**DOB:**	**LOCATION.**
**MRN:**	**INPATIENT**

HEIGHT: ies **WEIGHT** ounds **BMI:**

REFERRING PHYSICIAN:
SUPERVISING PHYSICIAN:

MEDICATIONS:

1. **Past 24 Hours:** Lipitor, Baclofen, Heparin, Neurontin, Fentanyl, Tylenol
2. **Held:** N/A

ALLERGIES: stadol,tetracycline

Hours since last caffeine intake: 24

REASON FOR TEST: Possible Ischemia

INDICATIONS FOR TEST: Known CAD, Shortness of Breath/Dyspnea, Hyperlipidemia, Abnormal Calcium Score, Hypertension, Positive Family History, Abnormal ECG, Atypical Angina

REASON FOR PHARMACOLOGIC STRESS: Pet Imaging

PROCEDURAL NOTE: An intravenous line was inserted and an infusion of normal saline was started. A CT scan was acquired for attenuation correction of the rest image (13 mA, 100 kVp, 2.7 secs) during end-expiration breath-holding, ECG-gating and dose modulation. This was followed by infusion of 35.1 mCi of Rb-82, 60 to 100 seconds after which resting images were acquired in list mode with ECG-gating. Following rest imaging, A total of 0.4mg regadenoson was injected intravenously over 10 seconds at a dosage of 0.08mg/ml immediately followed by a 5ml normal saline flush.At 2 minutes, 35.1 mCi of Rb-82 was infused. Peak stress images were acquired in list mode with ECG-gating, starting 30 seconds after the the beginning of the infusion of Rb-82 and continuing for 5.5 minutes. A CT scan was acquired for attenuation correction of the stress image (13 mA, 100 kVp, 2.7 secs) during end-expiration breath-holding. A coronary calcium score was acquired with 3 mm thick slices using a 3 mm slice to slice step.

Calcium Score Protocol: High-resolution, ECG-synchronized Computed Tomography (CT) of the heart and coronary arteries was performed using a Multi-Detector Computed Tomography (MDCT) scanner. Each slice acquired was 3 mm thick using a 3 mm slice to slice step. The patients average heart rate was 57 bpm and varied over a range of 3 bpm. There were no image artifacts..

CLINICAL RESPONSE: The heart rate at rest was 57 beats per minute. After the regadenoson injection the heart rate was 104 beats per minute. The blood pressure at baseline was 146/65 mmHg. At the end of the regadenoson injection it was 125/69 mmHg. The patient experienced the following symptoms during the test: nausea, back pain, chest discomfort, arm discomfort, leg discomfort.

© 2021 Patel KK et al. *JAMA Network Open.*

ELECTROCARDIOGRAPHIC FINDINGS: The resting 12-lead electrocardiogram showed sinus rhythm, poor progression of R wave across the anterior leads but is otherwise unremarkable. Following Regadenoson stress, there were no significant ECG changes. No arrhythmias were detected.

SCINTIGRAPHIC FINDINGS: The tomographic images show a moderately sized, moderate to severe reversible defect anteriorly and anterolaterally. The left ventricle does not dilate with stress. The gated tomograms show normal wall motion and thickening in all areas. The LVEF at rest is 76%, rising to 82% at peak stress.

Coronary Artery Calcium Score: The total Agatston score is 57.4. The coronary calcium score was 23 on 8/19/2014.

(This is a limited CT scan of the chest for evaluation of the coronary artery calcification only and is not intended for any other purpose.)

IN SUMMARY, the clinical, electrocardiographic, and scintigraphic findings in this 60-year-old female being evaluated for ischemia using regadenoson are as follows:

1) Clinical response: Non-Diagnostic (Regadenoson)
2) Electrocardiographic response: Non-Ischemic
3) Scintigraphic response: Ischemic

The combined test findings indicate the following:

1. A moderately sized area of moderate to severe ischemia probably in a large diagonal vessel.
2. Computer quantitation identifies 11% of the left ventricle as being ischemic.
3. Normal myocardial perfusion reserve (global MPR is 2.9).`
4. Normal left ventricular systolic function, LVEF at rest is 77%, rising to 85% at peak stress.
5. Coronary artery calcium scan does show evidence of coronary calcification. The Agatston score is 57.
6. Compared to the prior study, the appearance of a perfusion abnormality is a new finding.
7. Test results were verbally communicated on date of dictation at approximately 1600 hours.

[illegible]

[illegible]

4330 Wornall Roau, Suite 2000
Kansas City, MO 64111
Phone: 8169311883
Fax: 8169316362

12/12/2018 3:55:00 PM
I: AW 12/12/2018 4:29:58 PM
Time Test Completed: 10:50:00 AM
Electronically Approved: 12/12/2018
5:04:38 PM

Coronary Artery	# of Lesions	Volume Score (mm^3)	Mass Score (mg)	Agatston Score
Left Main	0	0	0	0
LAD	5	23.4	7.44	31.9

© 2021 Patel KK et al. *JAMA Network Open.*

eFigure 2. Sample Design of Patient-Centered Tool Sent as an Addendum to the Structured Patient Report

FRONT

Patient Name: __________ DOB: ___________ Test Date: _________

YOUR STRESS TEST RESULTS AND WHAT THEY MEAN

YOUR PERSONALIZED RISK BASED ON YOUR RISK FACTORS, SYMPTOMS AND STRESS TEST

Your personal risk is a combination of findings on your ECG, scan images at rest and stress, pump function and blood flow to the heart at rest and stress. It is important to combine your stress test results with other risk factors, such as your age, weight, high blood pressure or diabetes among others.

You should talk about your stress test results with your doctors. This paper is meant to help you understand your results better and help guide your discussion with your doctor, and does not replace your doctor's advice.

You have a **<u>HIGH RISK</u>** OF SIGNIFICANT BLOCKAGE IN YOUR HEART ARTERIES

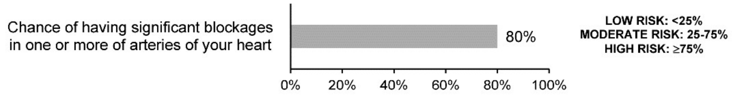

8 in 10 patients with similar results to yours had significant blockages in one or more of the arteries that supply your heart muscle. This puts you at a **HIGH** risk for having significant blockages in one or more of the arteries of your heart. ***Based on these results, your doctor may recommend further testing such as angiography (injecting contrast dye into the arteries that supply your heart muscle to identify and possibly treat any major blockages), and potentially treat with stents or bypass surgery.***

You have a **MODERATE-HIGH RISK** OF HEART-RELATED EVENTS

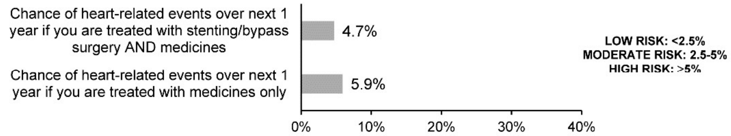

When patients with similar results to yours were treated with medicines listed below, *6 in 100 patients* had heart-related events in the next 1 year, this risk was reduced to 5 in 100 with stenting or bypass surgery if indicated. This puts you at a **MODERATE-HIGH** risk for having any heart-related events such as heart attack or death.

Based on your risk factors identified with this study, your doctors may discuss the following things with you (if they apply) to improve your heart health and reduce your cardiac risk.

- Starting medicines such as: baby aspirin, cholesterol medicine (statin) to reduce your cardiac risk.
- Starting heart medicines (such as beta-blocker (eg: metoprolol, carvedilol), ACE inhibitor (eg: lisinopril, enalapril), calcium channel blocker (eg: amlodipine among others) which lower your blood pressure, decrease the strain on your heart and help reduce your cardiac risk.
- Risk factor modification including: heart healthy diet (vegetables, fruits, nuts, legumes, fish and whole grains), regular exercise (≥150 min/week of moderate intensity or ≥75 minutes/week of vigorous physical activity), adequate control of high cholesterol, diabetes, blood pressure (BP goal <130/80mmHg), stopping smoking.

BACK

Patient Name: __________DOB: ________Test Date: ______

YOUR NUCLEAR STRESS TEST RESULTS

1. **CALCIUM SCORE** (May not apply to all stress tests): *estimates the amount of calcium deposits in your heart arteries, which can be a sign of blockage. It does not tell, however, if the blockages are severe or not. (Normal=0)*

   ☑ Moderate calcification (101-399, Your value was 312).

2. **MEDICINE STRESS PARAMETERS (May not apply to all stress tests):** *Some patients require use of medicine to simulate the conditions of exercise where your heart requires increased blood flow. Common medicines used for this purpose are regadenoson (Lexiscan), dipyridamole, adenosine and dobutamine. The activity of these medicines is usually short-lived only for the duration of the stress test. You may have received a reversal medicine if you had any severe side-effects or symptoms with it.*

   ☑ You required medicine to increase blood flow to heart, either because you are unable to exercise, or because you are having a PET stress test because you have other cardiac risk factors.

   ☑ You did not have chest pain or any high-risk EKG changes with stress.

3. **REST AND STRESS CARDIAC IMAGE SCAN (MPI, myocardial perfusion imaging):** *If a part of your heart is not getting enough blood supply when your heart is stressed, it may be seen as an abnormal defect on the image scan, and referred to as "ischemia" or "reversible perfusion defect". If a part of your heart has some permanent heart muscle damage, likely due to a prior heart attack, it is present both on rest and stress images, and is referred to as "infarct" or "fixed perfusion defect". (Normal value= 0%)*

   ☑ Your resting cardiac image is **<u>abnormal</u>**, suggesting possible prior damage to the heart. This is affecting a **small portion** of your heart muscle.

   ☑ Your stress cardiac image is **<u>abnormal,</u>** suggesting some blockages in the blood supply to the non-damaged areas of the heart. This is affecting a **significant portion** of your heart muscle. This is a high-risk marker on your test.

4. **YOUR HEART PUMP FUNCTION (EJECTION FRACTION):** *The amount of blood in the main pumping chamber that is pushed to the body with each heartbeat. (Normal: >50-55%)*

   Your heart pump function is **<u>normal</u>** at rest.

   Your heart pump function is **<u>normal</u>** with stress.

   You had a **<u>mild decrease</u>** in heart pump function with stress. This is a high-risk marker on your test.

5. **BLOOD FLOW TO THE HEART (MYOCARDIAL BLOOD FLOW RESERVE, MBFR: only applicable for PET)** *This number estimated by a PET stress test, indicates the factor by which the blood flow to the heart increases with stress. The normal value is usually 2 or greater, suggesting an increase of blood flow by more than 2 times at stress, compared to rest.*

   ☑ There is a decrease in blood flow to your heart when it is stressed, it is in the mild to moderately abnormal range (your MBFR value= 1.73). This is a high-risk marker on your test.

To obtain additional information about how the individual components of your stress test affect your risk and what you can do about it, please go to www.saint-lukes.org/**.***

© 2021 Patel KK et al. *JAMA Network Open.*
